# Reactions, Adaptation, and Support Needs of Siblings of Children with a Life-Limiting Disease: The Parents’ Experiences

**DOI:** 10.3390/children13050620

**Published:** 2026-04-30

**Authors:** Torun Marie Vatne, Elise Olsen Pedersen, Hanne Cathrine Lie

**Affiliations:** 1 Norwegian Centre for Rare Diseases Unit Frambu, Sandbakkvn 18, 1404 Siggerud, Norway; 2 Department of Behavioural Medicine, Institute of Basic Medical Sciences, Faculty of Medicine, University of Oslo, 0371 Oslo, Norway; eliseolsenpedersen@gmail.com (E.O.P.); h.c.lie@medisin.uio.no (H.C.L.)

**Keywords:** siblings, life-limiting disease, psychological adaptation, psychological reaction, support needs, family-centred care

## Abstract

**Highlights:**

**What are the main findings?**

- Siblings of children with life-limiting disease show both adaptation and significant emotional strain in relation to their family life with a seriously ill sibling.
- Sibling support occurs across multiple everyday contexts, such as from assistants or through aids in the family home, from family, friends, health care, and school. However, support provided to siblings is often uneven, fragmented, or insufficient.

**What are the implications of the main findings?**

- Siblings must be systematically included in pediatric palliative care from an early stage of the child’s life-limiting illness and palliative care.
- Care needs to be family-centered, provided within the everyday life of the family and coordinated across contexts.

**Abstract:**

Background/Objectives: Siblings of children with the life-limiting disease Metachromatic Leukodystrophy (MLD) are growing up witnessing rapid disease progression and death, affecting development and psychological wellbeing. Family-centred palliative care should include sibling support, but research on sibling needs is scarce. This semi-structured interview study aims to describe parents’ perceptions of siblings’ behavioural adaptation, emotional reactions, and support needs. Methods: Seven parents recruited from a national resource centre for rare disorders participated in semi-structured interviews that were audiotaped and transcribed. Content analysis was used to identify recurrent themes. Results: Siblings were described as more mature, patient, caring, appreciative, and sociable than other children due to life with the ill child. Expressions of love and concern, fear, sadness and sorrow, anger, and hope were described. Parents described how sibling support implies strengthening close family relations and providing resources at home, external support, and information. Conclusion: This study shows that sibling support involves all instances surrounding the family and ill child, and that a family-centred, trauma-sensitive approach is needed.

## 1. Background

Metachromatic leukodystrophy (MLD) is a neurological disease, causing loss of functions and early death in children. There are three forms of MLD, two with a childhood onset. Late infantile MLD has an onset before 2.5 years of age with a life expectancy of 3–4 years. It presents with difficulties walking, failure to reach motoric milestones and loss of deep tendon reflexes. With progression, CNS symptoms such as spasticity, cerebellar outcomes, and visual impairments appear. Juvenile MLD has a debut between 2 and 16 years of age, with a life expectancy of 10 to 20 years. Early symptoms are changes in behaviour and cognition, psychiatric symptoms, poor fine motor skills, and attention deficits. There are no cures or life-prolonging treatments for MDL but new treatments such as gene therapy can slow the progression of the disease [1,2].

Having a child diagnosed with a life-limiting disease (LLD) is a family crisis. Parents report high levels of distress and low quality of life [3,4]. LLD involves changes in living arrangements, working status, and hours devoted to caregiving for parents [5]. For siblings, life becomes substantially altered as the illness progresses, parents are distressed and the family life changes. Perceptions of the disease, its impact on daily life and emotional consequences greatly affect siblings of children with LLD [6], and clinicians should help parents remain sensitised to their needs [7].

In general, siblings of children with disabilities have increased risk of mental health problems compared to siblings of healthy children [8], with illness severity [8], impact on daily family functioning [9], and distress in parents [10] identified as specific risk factors. Still, a study of siblings of children with LLD found normal levels of psychosocial wellbeing [7]. Coping strategies may explain this finding, as siblings may hide distress and cope with emotions alone [7,11]. Siblings of children with LLD have been found to diminish their own needs, inhibit difficult feelings in daily life and take a more adult-like role in the family [7].

In Norway, children with LLD often live at home, and family-centred care aiming to increase the well-being of all family members [12] is emphasised in the national guidelines for paediatric palliative care (PPC) [13]. Family-centred PPC is defined as a seamless continuity in addressing patient, family, and community needs related to terminal disease through interdisciplinary collaboration [14]. It is recommended that services are trauma-informed, screen siblings for trauma exposure, provide families with resources about trauma, and ensure continuous care [15]. Research on support to siblings of children with LLD is scarce, but interventions cognizant of the trajectory of the illness as well as the family, school, and social contexts are highlighted as important [6].

As far as we know, no previous studies have explored parents’ perceptions of siblings’ emotional reactions and adaptation to life with a sibling with MLD. Nor have we found research on how to best provide trauma-informed family-centred support to siblings of children with MLD. This study aims to expand current knowledge by exploring:

(1) What kind of emotional reactions and behavioural adaptation do parents observe in daily life among siblings of children with MLD?

(2) What do parents see as good ways of supporting siblings of children with MLD in daily family life?

## 2. Materials and Methods

### 2.1. Study Design

This is a qualitative interview study using inductive content analysis. The study was approved by the National Centre for Research Data 12 June 2020 (#940820). All participants gave written informed consent.

### 2.2. Sample

Parents of children with late infantile or juvenile MLD who also had typically developing children were identified in the patient record of Frambu Center for Rare Disorders and invited to join the study. Parents of children who had died within the last five years were also invited. Of the 24 families invited, nine parents (representing five families) consented to participate, of which two parents later withdrew.

Participants included five mothers and two fathers from five families; three were bereaved. The families had one or two healthy siblings. In families where the child with MLD was alive, the ages of the siblings (*n* = 3) were two months, three years, and 13 years at the time of the interviews. In the bereaved families, the siblings (*n* = 5) were 4–18 years (mean 9.6 years) at the time of the child’s death. Two families had children with late infantile MLD, two families had children with juvenile MLD, and one family had two children with late infantile and juvenile MLD.

### 2.3. Data Collection

Parents were individually interviewed by phone by two of the authors, both females, following a semi-structured interview guide (see Table 1) developed based on a literature review, expert knowledge, and discussions with parents of children with LLD during a residential family course at Frambu. One interviewer was a clinical child psychologist with long experience working with families of children with MLD, and the other was a medical student experienced with the patient population through their own family history. The participants were informed about the interviewers’ experiences with MLD. The interviews lasted from 30 to 90 min. Audio recordings of the interviews were anonymously transcribed ad verbatim. All names in the transcripts were replaced with pseudonyms. Field notes were not made. Transcripts were not returned to participants for correction.

### 2.4. Data Analysis

Data was analysed inductively using conventional content analysis [16] primarily by author EOP, but regularly discussed with the other authors. The analysis followed the four steps of Malterud’s systematic text condensation [17]. (1) Transcripts were reviewed several times while noting content and possible themes, ending with seven preliminary themes. (2) Interviews were reviewed again, and the themes were reduced to five. Utterances corresponding to the five themes were coded and discussed. (3) The utterances coded within each theme were reviewed, coded in sub-themes based on abstracted meaning, and text condensates were developed for each sub-theme. (4) Analytic texts of each sub-group were developed using the condensates and quotes. Validity was ensured through repetitive discussions of the codes and condensates among the authors.

## 3. Results

### 3.1. Children’s Reactions and Adaptation to Living with a Child with MLD

**Figure 1:**
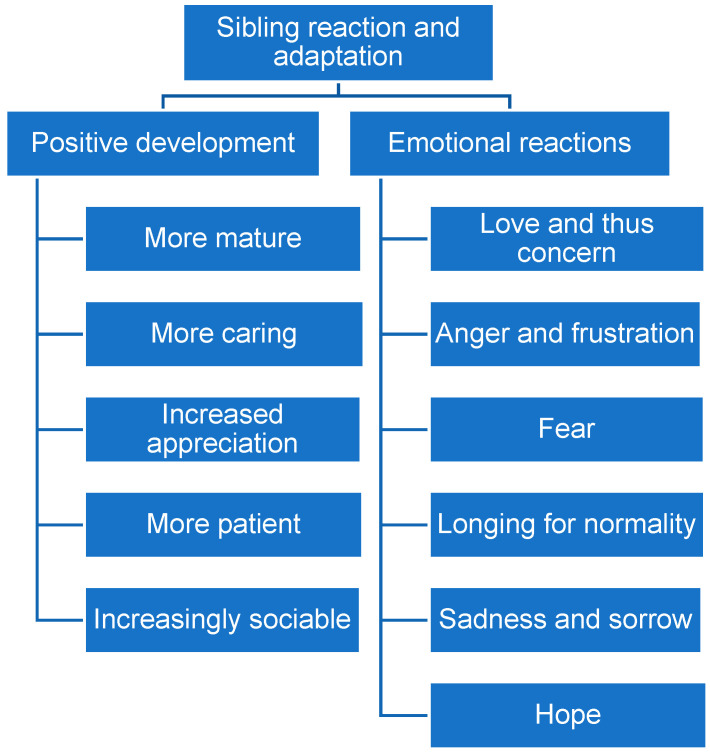
Themes and subthemes in parents’ descriptions of TD siblings’ emotional reactions and behavioural adaptations.

Participants’ descriptions covered two main themes: Positive development and emotional reactions, both with subthemes (Figure 1).

#### Positive Development

When asked about adaptation, the participants in this study described how siblings adapted to the family situation in a positive way. Siblings were described as *more mature* due to their life experiences: “I think his ‘backpack’ is heavier compared to other kids because of all the negative stuff when you have a sick sibling… but the positive sides are much greater”.

Becoming *more caring* because of life with a child with MLD was described: “She is really into him and cares about his wellbeing, and whether people around him are competent and good enough for him”. Siblings also showed care for peers and parents: “It’s important not to be too emotional because if you do, she takes on this comforting role which she is not supposed to have”.

Siblings’ *increased appreciation* for diversity was also described: “He is really understanding, and he knows people are different”.

Siblings could become *more patient* due to experiences in daily life: “He is actually quite patient too. He must wait a lot, wait, and accept a lot… we (parents) have so many things we have to do”.

Siblings were described as *increasingly sociable* because of all the health care personnel (HCP) and assistants at home: “He is extremely social and not a bit shy, and I think it’s because there is a lot of people in our house all the time, and often new people”.

### 3.2. Emotional Reactions

Varied emotional reactions in siblings were described. Reactions and adaptations could reflect parental coping: “I feel like they (siblings) were attentive to me and my reaction. Like they absorbed how we (parents) were doing emotionally, especially right after diagnosis, I think they noticed that there were a lot of things that were not good.”

Efforts to reduce negative reactions were described. “We were very focused on the fact that we just have to accept this, it’s nature… it’s okay to be sad and it’s okay to be angry, but not everything helps. Focused a quite a lot on the positive Simon (child with MLD; cMLD) brought into the family.”

*Love, and thus concern*, for the child with MLD was described: “She is really into Peter (cMLD) and gives him a lot of cuddles and love. Really aware of everything we do with him and quick to tell us if she thinks we lift him in a wrong way…”.

*Anger and frustration* were described: “I know they are so angry at this disease, and frustrated because the doctors couldn’t fix it…”. Anger could be expressed when TD siblings had to wait for parental attention due to the sick child.

A constant *fear* for medical emergencies was evident in siblings’ increased attentiveness: “Especially if he swallows wrong and cough, she has got him on the radar immediately. Even if she is in this distant iPad world, she is right there at once”. The fear could also inhibit the sibling relation: “They feel unsafe and are hesitant to spend time with her (cMLD)”.

Fearful reactions to sounds from equipment, alarms, or emergencies were described. Siblings could run away, cover sounds with music, or hide, and supporting them was difficult: “It’s difficult for them to see her in pain. In those situations, it is unfortunate because you get so into the one that’s sick and have to deal with the others in the aftermath”.

A *longing for normality* in siblings was described, in small siblings, evident in their comments: “He comments obvious facts like ‘Simon can’t walk’ and how Simon differs from other kids”. The longing of older siblings was seen as reluctance to inform others about the diagnosis or avoiding “normal” families: “She hates spending time with other families with two healthy children… She gets very jealous and sad when she sees the fun and the relationship they have”.

*Sadness and sorrow* were described, and to be triggered by many situations, to come in waves, and to affect memory and concentration and result in poor school performance: “It has been difficult for them to talk about him (cMLD) at school. When they do, they often cry”.

However, siblings could carry *hope*: “She has a hope that everything is going to be ok someday… recently she said ‘dad, are you absolutely sure that Peter is going to die’”.

### 3.3. Siblings’ Need for Support

**Figure 2:**
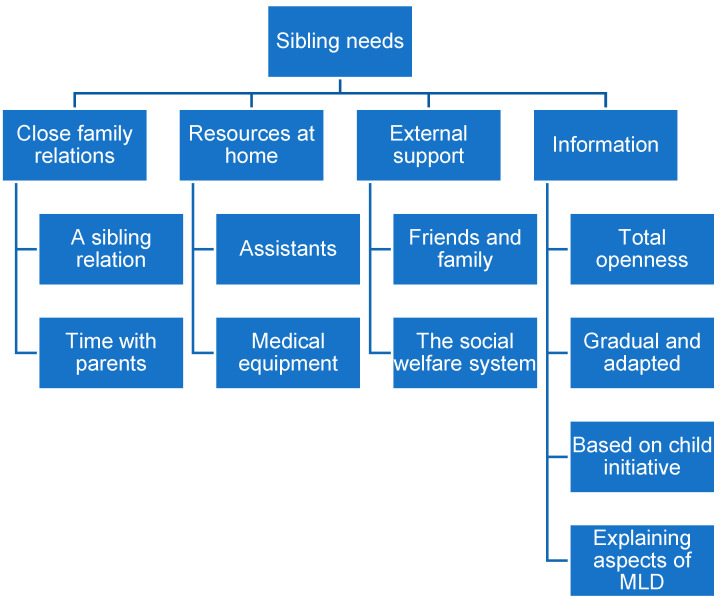
Themes and subthemes in parents’ descriptions of TD sibling support.

The participants’ descriptions of siblings’ need for support covered five main themes: close relations, resources at home, external support, and information about the illness, all with sub-themes (see Figure 2).

#### 3.3.1. Close Family Relations

Close family relations were described as a supportive factor in siblings’ lives. The need for a *sibling relation* was seen in efforts to engage in play: “If Jonas (cMLD) and Eric play with cars, Eric always put a car in Jonas’ hand so that it appears like he is driving”. Joint activities were seen as strengthening the relationship and were enforced by parents. One way was to set aside time: “I use to let him into the room with Jonas (cMLD) in the morning. In this way they have some quality time alone”. Another way was to assist: “If Eric (sibling) tries to say something to Jonas during play we answer for Jonas”.

Siblings adapted joint activities as the illness progressed: “He would lay down and talk and tell her (cMLD) all about his day at school”. Singing or reading was also described. Although siblings were described as best friends and playmates of the child with MLD, others struggled: “She says ‘I’m only with Peter (cMLD) for about five minutes or so… then I go to my room… I need you to help me to find something I can do with him every day’”.

*Time with parents* was described as important for sibling wellbeing: “I find it really important to be aware of him (sibling)… that he once in a while really needs parents as well’. Having a sick child could make parents more physically present in TD siblings’’ lives: “We (parents) were home when they left for school and there when they came back. Now (after child’s death), we are working again, they say ‘I wish you were home’.”

However, paying attention to siblings was described as difficult: “Jonas (cMLD) needs constant attention… and if we are alone, Eric gets little attention”. Although siblings loved time alone with parents, mixed feelings were described: “He thinks it’s fun, and has a good time, but he will ask for him (cMLD). He would rather like the whole family to be together”.

#### 3.3.2. Resources at Home

Participants described how resources provided due to the illness could enable sibling support, such as *assistants*: “The assistants have grown really fond of the two others (siblings) too, and they will just as well put them on the lap or help them”. Assistants also provided indirect sibling support by ensuring that parents were more available to siblings. However, the lack of stability in the team of assistants was a challenge.

*Medical equipment* could also meet sibling needs by strengthening opportunities for interaction: “We were offered a hospital bed, but we made a big round one that could be raised and lowered. The siblings loved to play there, and it became an environment that included them both”. New medical equipment could also stimulate communication about the illness with siblings (as described below).

#### 3.3.3. External Support

The participants described sibling support provided or desired. They wished that people would actively offer support and suggest activities with siblings rather than parents having to ask for it. Planned or spontaneous support from *friends and family* was seen important, enabling parents to spend time with siblings, or ensured some normality in daily life by helping siblings join leisure time activities: “Their aunt and uncle are really there for them, because when Ann (cMLD) is awake she has to be in my lap, … their aunt and uncle take them out on trips, do what we are not able to do.”

*Social welfare system* support was seen as important but varied greatly and could be lacking: “I feel like siblings’ existence is not taken into consideration at all… it’s just not good enough”. Others described great support: “The whole family became visible; everyone did their best to meet our needs and all doors opened”. A palliative team was described as a solution: “There should be a paediatric palliative team around us, but… neither we nor the kids, or anyone has got that”. The importance of sibling support “from day one” was expressed: “(…) someone that could have met him… played with him, … and tried to tell him all the stuff that I’m not able to explain”.

School support was often lacking: “The school nurse should have paid more attention. One visit, and then the years went by. MLD doesn’t pass after some months”. Schools well informed about sibling needs were important: “It’s good that the teacher knows that her brother is sick. That it could be the reason why she is very sad one day or hasn’t done her homework”.

However, some siblings refused the support offered because they did not want to “appear different”. Refusal of professional help was difficult for parents: “It is going to come to a point where we are not able to help her. She should talk to a professional about this, but so far, she does not want to”.

#### 3.3.4. Information

Participants described a need for honest information about MLD to siblings, but the perception of the right timing, the right amount, and what information provisioning strategies to adopt varied. Some aimed for *total openness*: “We chose to be open about everything from the start… they always got the same information as us…”. Total openness was motivated by believing that lack of information causes misunderstandings, advice from HCP, or a wish of being together in their grief: “We chose to tell her that it ends with death, mostly to give her the possibility to join us in the grief”. Some awaited genetic testing of siblings before providing all information, so they could reassure siblings that they were not going to get MLD themselves.

Some provided information in a *gradual and adapted* manner: “(…) I must inform him about the disease, but I will spare him from a lot. I will include the big lines but not get into details”. Attempts to prevent negative emotional reactions by adapting information and controlling one’s own emotions were described: “I am very keen to make all aspects of the diagnosis seem more harmless and not appear sad when I talk about Tom (cMLD)”. This was also related to sibling age: “I am really into not making the diagnosis scary, I inform in a way that doesn’t scare him. But when he’s older, I must tell him that this is a dangerous disease”. Information could be adapted to the sibling’s personality: “… some children may need a lot. I think it’s quite individual. You must be responsive to their need”.

Visible signs of the disease, new symptoms, or new medical equipment were described as opportunities for informing siblings: “When he was two years old, and still did not walk, they (sibling) tried to teach him. So, we had to tell them that there was no point trying to make him walk because he had this disease and would never be able to walk”.

Some only provided information *based on child initiative* to prevent fear and worry. Providing honest answers was described as important: “We had a deal that if they (siblings) asked if he was going to die, we would provide an honest answer, but they did not ask until right before (death)”. However, sibling’s ability to ask parents were also questioned: “If a child is very quiet and don’t dare to ask questions, they still might need information… that was the reason why we made sure they had several adults to talk to”.

A need for books or films to inform siblings about the medical aspects of MLD was described: “… having aids to use when we talk about the disease at home, like books and movies, not necessarily focusing on the sad parts but on what actually happens”. Including siblings during hospital visits provided opportunities for information: “We had a standing offer at the pediatric unit to come and talk with the pediatrician”.

Some particularly important, but difficult, topics for conversation with siblings were described. One was the progressive nature of MLD: “It’s important that she knows that this illness isn’t a static state but progressive. That in one moment everything may be alright and in the next we must rush to the hospital”. Death was another important and difficult topic: “(…) To have an understanding of death… I feel it’s important to inform him, even if he doesn’t ask. It must be learned and understood… Some die old and some die young.” Talking about death in general was described as a starting point, to make siblings able to ask questions, but aids were needed: “I would like to have books that I could read to make him understand what it’s like when a person dies”.

## 4. Discussion

### 4.1. Discussion

Behavioural adaptation in siblings of children with MLD was described by parents in this study. The maturity, patience, care, sociability, and appreciation for diversity described in siblings are in line with results of previous sibling research [7]. Although parents described the adaption as positive, it is important to remember that siblings’ behaviour also may reflect unhealthy coping efforts [7] and should be understood keeping the family crisis in mind [15]. Siblings being ‘more mature’ may reflect that they take on responsibility in response to adversity at the cost of age-appropriate behaviours and developmental experiences. Similarly ‘being patient’ may be a necessity shaped by constrained circumstances without other option but to adapt, comply, or suppress own needs.

Parents in this study described various negative emotional reactions among siblings. As siblings’ risk for developing psychological problems are elevated [8] and parents are found to be good reporters of sibling’s emotional state [18], the results indicate that providing emotional support to siblings of children with MLD is important.

When facing difficult topics parents described to maximise positive feelings to reduce the emotional burden on siblings. Emotional communication with siblings can be difficult for parents [19,20]. Thus, family communication should be a topic in family-centred PPC and communication training for parents shows promising results [21].

Parents called for sibling support from the system from the time of diagnosis. In Norway, HCP are obliged by law to ensure such support [22]. If PPC plans involve the siblings from the beginning, this may facilitate siblings accepting help later on.

In line with results of previous research on families of children with genetic disorders [23], parents described difficulties informing siblings about the disease. By assisting parents, HCP may prevent anxiety in siblings [24,25]. The information practices described in this study were the results of consideration of aspects of the illness, the sibling and family factors. This is line with the current professional opinion emphasising that there is no “black-and-white” answer when disclosing a life-limiting illness to children [26]. The progressive nature of MLD was described to provide a natural pace in the information process. Thus, HCP support should follow the family’s pace throughout the disease trajectory.

Visiting the hospital was described as an opportunity for sibling support from HCP. Previous research has also described how HCP may prepare children for procedures through information, gradual exposure, or medical play [18,27,28]. Trauma-informed sibling care at the hospital may involve preparing siblings for symptoms, seizures, and procedures (e.g., use of suction aid).

For children with MLD, multiple medical emergencies are inevitable. Siblings’ anxiety related to these situations were described by parents, but proper handling found difficult. The health and social welfare system could foster post-trauma recovery in siblings by acknowledging their presence and provide information and advice parents on how to support siblings in the aftermath [29]. A crucial part of PPC is developing family-centred care plans as the disease progresses [30]. Our results speak to the importance of specifying care of siblings in times of medical emergencies or death in these plans.

The importance of family relations for siblings’ wellbeing was emphasised by the parents in this study. Although the Norwegian government advocate for a family perspective when social services are provided [31], parents in this study described how lack of, and instability in service delivery, limited siblings’ possibility for joint family activities, time with parents, or friends. Family-centred PPC, as advocated in the national guidelines [13], should involve delivering care to the child with MLD in a manner and scope that also keep the needs of siblings in mind.

Sibling relation and joint activities, facilitated by aids and assistance, were described as important, but could be difficult as illness progressed. This aligns with previous research, and methods to teach interaction strategies have been tested with good results [32].

Social network was important for siblings as it provided time with parents, communication partners, and normal leisure activities. Organising network meetings with family and friends, with psychoeducation and discussions of possible support, have been found to be an efficient way of providing such support [33,34]. Strengthening the network surrounding siblings could also involve day-care or school as sibling reactions were not always met with understanding in this setting. Trauma sensitive schooling and day-care could be ensured by informing personnel about experiences and emotional reactions of siblings and good ways to support these children in everyday life.

This study has limitations. First, the participants were recruited through a competence centre and thus only represented families who had received their services. This may have affected the participants’ experience of sibling support. Second, the sample was small and heterogenous in terms sibling age. Nuances related to specific age-related needs may therefore have been lost. Finally, the interviews were conducted by phone which may have affected the relationship between the interviewer and the participant and thus the quality of the data.

### 4.2. Conclusions

We found that the reactions and adaptation of siblings were related to family, disease, and social factors. Further it shows how sibling support involves all instances surrounding the family and the child with MLD, and that a family-centred, and trauma-sensitive approach, including explicit attention to sibling support, is needed.

## Figures and Tables

**Table 1:** Interview guide.

1. What challenges do you experience that X (sibling’s name) has had during the course of the illness?
2. What kind of support has X received during the course of the illness?
3. Which changes have you seen in X related to what the family is/has been going through?
4. What do you think X thinks about his/her sibling’s diagnosis?
5. How did X come to know what he/she knows now?
6. What do you think TD siblings need of information about their sibling’s diagnosis?
7. How do you feel about talking to X about the diagnosis?
8. In an ideal world, what support do you think siblings of children with neurodegenerative disease should receive?

## Data Availability

The datasets generated during and/or analysed during the current study are not publicly available due to protection of the participants’ privacy but are available from the corresponding author on reasonable request.
